# Cholecystokinin A Receptor (CCKAR) Gene Variation Is Associated with Language Lateralization

**DOI:** 10.1371/journal.pone.0053643

**Published:** 2013-01-14

**Authors:** Sebastian Ocklenburg, Larissa Arning, Wanda M. Gerding, Jörg T. Epplen, Onur Güntürkün, Christian Beste

**Affiliations:** 1 Institute of Cognitive Neuroscience, Biopsychology, Ruhr-University Bochum, Bochum, Germany; 2 Department of Human Genetics, Ruhr-University Bochum, Bochum, Germany; Baylor College of Medicine, United States of America

## Abstract

Schizophrenia is a psychiatric disorder associated with atypical handedness and language lateralization. However, the molecular mechanisms underlying these functional changes are still poorly understood. Therefore, the present study was aimed at investigating whether variation in schizophrenia-related genes modulates individual lateralization patterns. To this end, we genotyped 16 single nucleotide polymorphisms that have previously been linked to schizophrenia on a meta-analysis level in a sample of 444 genetically unrelated healthy participants and examined the association of these polymorphisms with handedness, footedness and language lateralization. We found a significant association of the cholecystokinin-A receptor (*CCKAR*) gene variation rs1800857 and language lateralization assessed using the dichotic listening task. Individuals carrying the schizophrenia risk allele C of this polymorphism showed a marked reduction of the typical left-hemispheric dominance for language processing. Since the cholecystokinin A receptor is involved in dopamine release in the central nervous system, these findings suggest that genetic variation in this receptor may modulate language lateralization due to its impact on dopaminergic pathways.

## Introduction

Schizophrenia is a complex psychiatric disorder that is likely to be caused by a combination of environmental and genetic factors [1]–[5]. Several studies have shown that schizophrenia is associated with atypical handedness and language lateralization. For example, Sommer et al. [6] meta-analyzed 16 studies that investigated handedness in schizophrenic patients and healthy controls and found that the patients had a higher prevalence of non-right-handedness than the controls. Comparable results were also obtained by a more recent meta-analysis by Dragovic and Hammond [7], who analyzed 42 studies that investigated handedness in schizophrenic patients. These authors also found a general leftward shift in handedness distribution, with a significantly greater prevalence of both left- and mixed handedness in schizophrenic patients than in healthy control participants. Language lateralization is a trait often assessed with the dichotic listening family of tasks, with the most widely used paradigm being the classic consonant vowel dichotic listening task [8]–[10]. In this task, participants wear headphones and two different short consonant–vowel syllables (e.g. BA or DA) are presented at the same time, one to each ear. After stimulus presentation, participants have to report the stimulus they heard best. Most individuals show a so-called right-ear-advantage in this task, reflecting a left-hemispheric language dominance [9]. Sommer et al. [6] meta-analyzed ten studies investigating language lateralization in schizophrenic patients with this and other types of the dichotic listening task and found that when only the consonant-vowel and the similar fused-word form of the dichotic listening task (who are thought to be the most accurate measure of language dominance, see [6]) were analyzed, schizophrenic patients showed a significantly decreased right-ear advantage compared to healthy controls. Subsequent research showed that especially those patients who frequently experience auditory hallucinations [11] or are actively hallucinating during testing [12] show a reduced right-ear advantage, while young and stable patients that do not experience hallucinations sometimes do not show such a reduction [13]. Based on these findings, Collinson et al. [14] concluded that the presence of positive symptoms and particularly of auditory hallucinations are an important determinant of whether or not a schizophrenic patient experiences dichotic listening deficits. Collinson et al. [14] also reported that the experience of dichotic listening deficits in schizophrenic patients is linked to structural changes in the temporal cortex. They found that schizophrenic patients who did not show the typical right-ear advantage in the dichotic listening test also showed a reduction of left temporal lobe volume when compared with patients with typical dichotic listening results and healthy controls.

However, despite the multitude of studies reporting atypical handedness and language lateralization in schizophrenic patients, the molecular mechanisms underlying these functional changes are still poorly understood. Therefore, the aim of the present study was to examine the role of schizophrenia susceptibility genes for handedness and language lateralization. To this end, we genotyped 16 single nucleotide polymorphisms (SNPs) that have previously been linked to schizophrenia on a meta-analysis level (see section 2.2 Genotyping and table 1 for details) in a sample of 444 healthy German students and examined the association of these SNPs with handedness and language lateralization. Since it has been reported that footedness is more strongly related to language lateralization than handedness [15] we also assessed this trait. Due to the link between dichotic listening deficits and the experience of positive schizophrenia symptoms, especially auditory hallucinations, we were particularly interested in the rs1800857 polymorphism in the cholecystokinin-A receptor (*CCKAR*) gene which has been shown to be related to these symptoms [16]–[18]. We hypothesize that those genotypes which have previously been shown to be related to an increased risk of schizophrenia are also linked to reduced or reversed limb preferences and language lateralization in our sample.

**Table 1:** Results of the three lateralization tests for the different schizophrenia-related polymorphisms.

	Handedness	Footedness	DL
CCKAR (rs1800857)	t_(440)_ = −0.37; p = 0.71	t_(440)_ = −1.75;p = 0.08	t_(440)_ = −3.39;p = 0.0008*
DAOA (rs2391191)	t_(441)_ = −0.35; p = 0.73	t_(441)_ = −1.00; p = 0.32	t_(441)_ = −0.41; p = 0.68
DAOA (rs778293)	t_(437)_ = 0.92; p = 0.36	t_(437)_ = −0.33; p = 0.74	t_(437)_ = −0.62; p = 0.54
DRD1 (rs4532)	t_(440)_ = −0.98; p = 0.33	t_(440)_ = −0.62; p = 0.53	t_(440)_ = 0.74; p = 0.46
DRD4 (rs1800955)	t_(436)_ = 1.45; p = 0.15	t_(436)_ = 1.33; p = 0.19	t_(436)_ = 0.97; p = 0.33
DISC1 (rs3737597)	t_(441)_ = −0.66; p = 0.51	t_(441)_ = 0.50; p = 0.62	t_(441)_ = 1.43; p = 0.15
GABRB2 (rs6556547)	t_(433)_ = 0.34; p = 0.74	t_(433)_ = 0.68; p = 0.50	t_(433)_ = −0.07; p = 0.95
MDGA1 (rs11759115)	t_(441)_ = −0.42; p = 0.68	t_(441)_ = −0.48; p = 0.63	t_(441)_ = 1.77; p = 0.08
NOTCH4 (rs3131296)	t_(441)_ = −0.49; p = 0.62	t_(441)_ = 0.09; p = 0.93	t_(441)_ = 0.49; p = 0.63
NRGN (rs12807809)	t_(437)_ = −0.60; p = 0.55	t_(437)_ = 0.71; p = 0.48	t_(437)_ = 0.35; p = 0.73
PGBD1 (rs13211507)	t_(441)_ = −0.99; p = 0.32	t_(441)_ = −0.52; p = 0.60	t_(441)_ = 0.29; p = 0.77
PLXNA2 (rs841865)	t_(440)_ = 0.04; p = 0.97	t_(440)_ = 1.66; p = 0.10	**t_(440)_ = 2.47; p = 0.01**
RELN (rs7341475)	t_(441)_ = −0.77; p = 0.45	t_(441)_ = −0.59; p = 0.56	t_(441)_ = 1.17; p = 0.25
TCF4 (rs9960767)	t_(441)_ = −0.50; p = 0.61	t_(441)_ = −0.42; p = 0.68	**t_(441)_ = 2.23; p = 0.03**
TPH1 (rs1800532)	t_(441)_ = 1.78; p = 0.08	**t_(441)_ = 2.44; p = 0.02**	t_(441)_ = 0.95; p = 0.34
ZBTB42 (rs3803300)	t_(440)_ = 0.06; p = 0.95	t_(440)_ = −1.07; p = 0.28	t_(440)_ = 1.32; p = 0.19

All comparisons that are significant on the p<0.05 level are given in bold letters. An asterisk indicates that the p-value is below p = 0.001325, the value needed to be still considered significant after Bonferroni correction.

## Experimental Procedures

### Participants

Four-hundred and forty-four (n = 444) healthy participants of Caucasian descent for at least two generations participated in the present study (257 women and 187 men). Participants had no history of any psychiatric or neurological diseases as assessed by experienced psychologists applying a psychiatric screening (sociodemographic questionnaires, BDI, HAMA, ASI) and were genetically unrelated. The mean age was 23.71 (range: 18–34 years). Participants were mainly university students, and all of them were native German speakers. In order to ensure that all participants had normal hearing capability (a prerequisite for the dichotic listening task) audiometric screening was performed before the start of the experiment. Hearing thresholds were screened at 750, 1500 and 3000 Hz using a MA 25 audiometer (MAICO Diagnostic GmbH, Berlin, Germany). Only individuals with hearing thresholds below 30 dB for all frequencies and inter-aural differences below 15 dB were included in the cohort.

### Ethics Statement

All participants gave written informed consent and were treated in accordance with the declaration of Helsinki. The study was approved by the ethics committee of the medical faculty of the Ruhr-University Bochum.

### Genotyping

DNA was isolated from exfoliated cells that were brushed from the oral mucosa of the participants. DNA isolation was conducted using a QIAamp DNA mini Kit (Qiagen GmbH, Hilden, Germany) according to the protocol provided by Qiagen. SNP's were genotyped using polymerase chain reaction (PCR) and differential enzymatic analysis was performed using the PCR restriction fragment length polymorphism method. Candidate SNPs were chosen from the SZGene database (http://www.szgene.org/ April 2010) which lists all genetic loci associated with schizophrenia. All polymorphisms included in the present study had at least one nominally significant meta-analysis result in SZGene. The following SNPs were selected: *CCKAR* rs1800857, *DAOA* rs2391191, *DAOA* rs778293, *DRD1* rs4532, *DRD4* rs1800955, *DISC1* rs3737597, *GABRB2* rs6556547, *MDGA1* rs11759115, *NOTCH4* rs3131296, *NRGN* rs12807809, *PGBD1* rs13211507, *PLXNA2* rs841865, *RELN* rs7341475, *TCF4* rs9960767, *TPH1* rs1800532, *ZBTB42* rs3803300. Further methodological details and primer sequences are available upon request.

### Limb preferences

Handedness was assessed using the Edinburgh Handedness Inventory (EHI, [19]) and footedness using the Waterloo Footedness Questionnaire (WFQ, [15], [20] ). For both questionnaires, a laterality quotient (LQ) was calculated according to the method of Oldfield [19] using the following formula: LQ = [(R−L)/(R+L)]×100. In this formula, R denotes the number of right-sided preferences indicated by the participant and L the number of left-sided preferences. The LQ ranges from −100 to +100, with positive values indicating preference for the right limb and negative values indicating preference for the left limb.

### Language lateralization

In order to assess language lateralization, we used a verbal dichotic listening task. The dichotic listening stimuli were syllable pairs consisting of two out of six different consonant-vowel syllables (/ba/,/da/,/ga/,/ka/,/pa/and/ta/) that were simultaneously presented (one syllable to each ear) using headphones. The syllables were matched for voice-onset time and had a standardized mean duration of 350 ms. They were presented at 80 dB, and the inter-stimulus interval was two seconds. Before beginning the task, participants were instructed to indicate as quickly and accurately as possibly which syllable they heard best on each trial. To this end, participants had to press one of six keys labeled with the six syllables on a customized response box. The task started with two practice runs of 12 trials each, which were excluded from later analysis. Subsequently participants performed four test blocks of 30 trials each, with each of the 30 possible dichotic combinations of the syllable pairs being applied once per test block. Thus, overall the task consisted of 120 test trials. To control for possible handedness effects, two of the test blocks were answered with the left and the other two with the right hand, in counter-balanced order. Moreover, to minimize effects of possible aural differences between the left and the right headphone channels, the headphones were reversed for two of the test blocks in counter-balanced order. In analogy to the LQ calculated for limb preferences, a dichotic laterality index (LI; [21]) was calculated using the following formula: LI = [(R−L)/(R+L)]×100. In this formula, R denotes the number of correct right ear responses and L the number of correct left ear responses. The LI ranges from −100 to +100, with positive values indicating that more stimuli perceived by the right ear were identified correctly (indicating left-hemispheric language dominance) and negative values indicating that more stimuli perceived by the left ear were identified correctly (indicating atypical right-hemispheric language dominance).

## Results

### Genotyping

All SNPs conformed to Hardy-Weinberg equilibrium (all p's>0.13), with the exception of the DISC1 rs3737597 and the DRD4 rs1800955 (both p's<0.05) as calculated using the online version of DeFinetti (http://ihg.gsf.de/cgi-bin/hw/hwa1.pl).

### Lateralization

Overall, participants had a mean handedness LQ of 71.07 (+/−2.42) and a mean footedness LQ of 43.48 (+/−2.01), both indicating that most participants preferred to use their right hand or foot. The mean dichotic listening LI was 39.53 (+/−1.55), indicating that on average participants had a right ear advantage, reflecting left hemispheric language dominance. The statistical analyses were performed assuming a dominant effect for each polymorphism. Thus, the heterozygous and the rarely observed homozygous genotypes were combined for statistical analysis. LQs for handedness and footedness and LIs for the dichotic listening task were analyzed using independent-samples two-sided t-tests (see table 1).

Overall, four effects reached significance at the p<0.05 level. For the *CCKAR* rs1800857 SNP, the combined TC/CC group (n = 113) had a lower dichotic listening LI (37.32+/−1.86) than individuals with the TT genotype (n = 329) (42.70+/−1.68; t_(440)_ = −3.39; p = 0.0008). For the *PLXNA2* rs841865 SNP the homozygous GG group (n = 311) had a lower dichotic listening LI (37.12+/−1.86) than the combined AA/AG group (n = 131) (45.63+/−2.74; t_(440)_ = 2.47; p = 0.01) and for the *TCF4* rs9960767 SNP the combined AC/CC group (n = 57) had a lower dichotic listening LI (30.77+/−3.85) than the homozygous AA group (n = 386) (41.03+/−1.67; t_(441)_ = 2.23; p = 0.03). A footedness effect was observed for the *TPH1* rs1800532 SNP. Here, the homozygous CC group (n = 160) had a lower LQ (37.19+/−3.84) than the combined CA/AA group (n = 283) (47.33+/−2.25; t_(441)_ = 2.44; p = 0.02).

**Figure 1:**
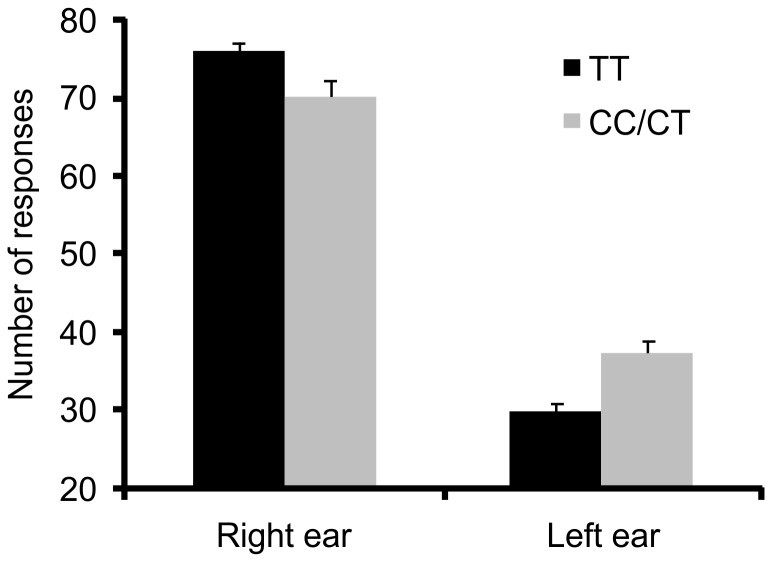
Association of CCKAR rs1800857 genotypes and performance in the dichotic listening task. The absolute number of right-ear and left-ear responses is shown (the maximal number of responses was 120). Error bars show standard error.

Since the effect of the *CCKAR* rs1800857 SNP for dichotic listening was the only effect that had a p-value below p = 0.001325 (the value needed to be still considered significant after Bonferroni correction) this effect was further investigated using a 2×2×2×2×2×2 repeated-measures' analyses of variance (ANOVA) with the three within-subjects factor ear (left ear, right ear), response hand (left hand, right hand), headphones position (normal, reversed) as well as the three between-subjects factors genotype (TC/TT, CC), handedness (left-handed, right-handed) and gender (male, female). Overall, the participants showed a clear right ear advantage (right ear: 73.31 responses ±1.08; left ear; 33.48 responses +/− 0.92) as indicated by a main effect of ear (F_(1,440)_ = 53.07; p<0.000001; η^2^ = 0.11). Moreover, the key interaction ear by genotype (see figure 1) reached significance (F_(1,440)_ = 16.87; p = 0.000048; η^2^ = 0.037), indicating that the combined TC/CC group had a reduced right ear advantage (left ear: 36.99 responses +/−1.60; right ear: 70.51 responses +/−1.86; Bonferroni-corrected post hoc test: p<0.001) compared to individuals with the TT genotype (left ear: 29.97 responses +/−0.94; right ear: 76.11+/−1.09; Bonferroni-corrected post hoc test: p<0.001). Moreover, the interaction ear by handedness reached significance (F_(1,440)_ = 10.48; p = 0.0013; η^2^ = 0.037), reflecting the well-known fact that left-handers are more likely to show a atypical right-hemispheric language dominance than right-handers are. All other main effects and interactions failed to reach significance, showing that the specific effect of CCKAR gene variation on performance in the dichotic listening task was neither affected by gender or handedness, nor by headphone position or the hand used to react.

## Discussion

The aim of the present study was to investigate the relevance of sequence variations in different schizophrenia-related genes for language lateralization and limb preferences. The analysis of 16 schizophrenia-related polymorphisms revealed an association between the *CCKAR* gene and language lateralization assessed with the dichotic listening task. While we observed the frequently reported right-ear advantage in the dichotic listening task [8], its extent was modulated by genetic variation within *CCKAR*, since we found a significant effect of the rs1800857 polymorphism in *CCKAR* on language lateralization. The polymorphism rs1800857 is located in intron 1 near the intron 1/exon 2 boundary (IVS1-5T>C) and could therefore have an impact on the mRNA splice acceptor site sequence. Yet so far, functional studies found no effect on the splicing efficiency of exon 2 in the *CCKAR* gene [16].

In line with our hypothesis that those genotypes which have previously been shown to be related to an increased risk of schizophrenia should be linked to reduced or reversed lateralization, individuals carrying at least one schizophrenia risk allele C [16] showed a reduced right ear advantage as compared to individuals with the homozygous TT genotype. This effect was due to both a decrease of the right ear score as well as an increase of the left ear score in individuals carrying the C allele compared to those without (see figure 1).

Functionally, *CCKAR* (located at 4p15.1 - p15.2) encodes the cholecystokinin A receptor which binds non-sulfated members of the cholecystokinin family of peptides [22]. The cholecystokinin A receptor modulates pancreatic function [23]. In the central nervous system it has been linked to dopamine release, especially within the mesolimbic system [16], [24]–[27]. Specifically, the cholecystokinin A receptor is thought to facilitate the functions of dopamine, and it has been shown that administration of the selective cholecystokinin A receptor antagonist devazipide prevents conditioned incentive learning, a dopamine-mediated learning process [28]. This relation to dopamine release made *CCKAR* an interesting candidate gene for schizophrenia risk, since a theory about the origins of schizophrenia postulated that it is the result of excessive dopaminergic activity in the brain [29]. While subsequent studies indicated that dysregulation in the dopaminergic system is not necessarily the neurochemical cause of schizophrenia, dopamine is the neurotransmitter operating the neuronal pathway that underlies positive schizophrenia symptoms [30], and indeed *CCKAR* had been linked to schizophrenia risk and especially positive psychotic symptoms [18] as well as the experience of auditory hallucinations [17], [31]. There is evidence suggesting that mostly schizophrenic patients that frequently experience auditory hallucinations have dichotic listening deficits [11]–[14]. Hence, these links between *CCKAR* and auditory hallucinations might explain why we found an effect of *CCKAR* on language lateralization. Moreover, the dopaminergic system has been linked to hemispheric asymmetries, with administration of the dopamine antagonist chlorpromazine leading to a reduction of emotional lateralization [32] and the nigrostriatal dopaminergic system playing a role in motor lateralization [33]. Thus, although the exact functional role of *CCKAR* rs1800857 polymorphism is not clear at present, our results nevertheless suggest that variation in *CCKAR* may modulate language lateralization potentially by influencing dopaminergic pathways.

While we found a significant association between variation in *CCKAR* and language lateralization, the effect size of the ear by genotype interaction indicated that only about 3.7% of the variance in the dichotic listening data could be explained by genetic variation. This rather low amount of explained variance and the fact that there was no association of the *CCKAR* polymorphism with handedness make an interesting implication regarding the inheritance of language lateralization and handedness. In contrast to theories that assume a common monogenic background for these two traits [34]–[35], the present findings suggest that handedness and language lateralization are partly independent and that language lateralization most likely is a polygenic trait. This view is also supported by a number of recent genetic and neuroimaging studies in humans [36]–[38] as well as by comparative studies [39]. Moreover, the assumption that language lateralization is a polygenic trait is further supported by a recent study that reported a significant association between the synonymous *GRIN2B* variation rs1806201 and language lateralization with a variance explanation of 2% [40]. Regarding the genetic determinants of language lateralization, the present study also showed that the rs841865 SNP within *PLXNA2* as well as the rs9960767 SNP within *TCF4* represent promising candidate polymorphisms for future studies. Moreover, our results indicate that the SNP rs1800532 within *TPH1* is a promising candidate polymorphism for the investigation of the genetic basis of footedness, while no effects regarding handedness have been observed.

In summary, the present study shows that variation in the *CCKAR* gene modulates language lateralization, with the schizophrenia risk allele C of the rs1800857 polymorphism being related to reduced functional asymmetry. Replication in independent healthy cohorts, and especially in schizophrenic patients with and without auditory hallucinations are needed in order to further validate the present results and would be particularly helpful in order to understand the complex relationship of *CCKAR*, dopamine and language lateralization.
